# Developing a Sensitive Platform to Measure 5-Methyltetrahydrofolate in Subjects with MTHFR and PON1 Gene Polymorphisms

**DOI:** 10.3390/nu14163320

**Published:** 2022-08-13

**Authors:** Mong-Liang Lu, Wei-Chi Ku, Nailis Syifa, Shu-Chin Hu, Chia-Te Chou, Yi-Hsio Wu, Po-Hsiu Kuo, Chun-Hsin Chen, Wei J. Chen, Tzu-Hua Wu

**Affiliations:** 1 Department of Psychiatry, Wan Fang Hospital, Taipei Medical University, Taipei 11031, Taiwan; 2 Psychiatric Research Centre, Wan Fang Hospital, Taipei Medical University, Taipei 11031, Taiwan; 3 Department of Psychiatry, School of Medicine, College of Medicine, Taipei Medical University, Taipei 11031, Taiwan; 4 School of Medicine, College of Medicine, Fu Jen Catholic University, New Taipei City 24205, Taiwan; 5 Department of Clinical Pharmacy, School of Pharmacy, College of Pharmacy, Taipei Medical University, Taipei 11031, Taiwan; 6 Pharmacy Department, Faculty of Health Science, University of Muhammadiyah Malang, Malang 65144, Indonesia; 7 Department of Pharmacy, Taipei Medical University Hospital, Taipei 11031, Taiwan; 8 Department of Public Health, Institute of Epidemiology and Preventive Medicine, College of Public Health, National Taiwan University, Taipei 10055, Taiwan; 9 Center for Neuropsychiatric Research, National Health Research Institutes, Miaoli 35053, Taiwan; 10 Master Program in Clinical Genomics and Proteomics, College of Pharmacy, Taipei Medical University, Taipei 11031, Taiwan

**Keywords:** 5-methyltetrahydrofolate, LC–MS/MS, MTHFR C677T, PON1 Q192R, schizophrenia

## Abstract

Inadequate levels of 5-methyltetrahydrofolate (5-MTHF) and the T variant of MTHFR C677T have been suggested to be associated with an increased risk of developing mental illness, whereas the PON1 SNP variant provides a protective role. However, reports validating the methodology for plasma 5-MTHF levels in schizophrenia patients are limited. A sensitive LC–MS/MS system using an amide column and calibration curve was determined by dialyzed human plasma, and applied to schizophrenia patients and healthy controls in Taiwan, and the differences between the subgroups were discussed. This analysis system meets regulation criteria, and the lower limit of quantification for 5-MTHF levels was 4 nM from 200 μL plasma, within 7 min. The mean plasma 5-MTHF levels in schizophrenia patients (n = 34; 11.70 ± 10.37 nM) were lower than those in the healthy controls (n = 42; 22.67 ± 11.12 nM) significantly (*p* < 0.01). 5-MTHF concentrations were significantly lower in male carriers than in female carriers (18.30 ± 10.37 nM vs. 24.83 ± 11.01 nM, *p* < 0.05), especially in subjects who were MTHFR CT/PON1 Q allele carriers. In conclusion, this quantitative system, which employed sensitive and simple processing methods, was successfully applied, and identified that schizophrenic patients had significantly lower levels of 5-MTHF. Lower plasma 5-MTHF concentrations were observed in male subjects.

## 1. Introduction

Methylenetetrahydrofolate reductase (MTHFR) converts 5,10-methylenetetrahydrofolate (5,10-MTHF) to 5-methyltetrahydrofolate (5-MTHF). Additionally, the Paraoxonase-1 (PON1) enzyme is responsible for homocysteine production, and 5-MTHF, which is demethylated from 5,10-MTHF by MTHFR, is the methyl donor for the remethylation of homocysteine by cobalamin-dependent methionine synthase to yield methionine. MTHFR SNP variants are linked to coronary atherosclerosis [1,2]. The TT genotype is responsible for the reduced activity of the MTHFR enzyme, which, in turn, leads to an increase in homocysteine concentrations [3,4]; however, MTHFR A1298C, but not C677T, was found to be associated with metabolic syndrome in schizophrenia patients [5]. PON1 SNP rs662 may have a role in metabolic syndrome [6,7], and those carrying its variants may exhibit reduced levels of PON1 activity and few responses to pharmacotherapy. Recent evidence supports the correlation between MTHFR SNP variants and an increased risk of developing mental illnesses, such as depression and bipolar disorders; however, knowledge regarding the implication of MTHFR SNP variants in schizophrenia is limited. 

In a 2014 study conducted in Egypt, the incidence of the TT genotype in the recruited population was significantly higher in 103 schizophrenia groups (OR 4.9; 14.9% vs. 3.4%) than in 149 control groups [8]. Another study recruited 330 healthy controls (2017), and MTHFR C677T mutations were associated with increased homocysteine levels and levels of erythrocyte folate, whereas plasma folate levels were slightly reduced [9]. The reduction of MTHFR enzyme activity, as well as the DNA methylation process, was later proposed to have significant impacts in schizophrenia [10]. However, there is little evidence that resolves the question of whether genetic variants in MTHFR C677T impair the efficacy of MTHFR in converting 5,10-MTHF to 5-MTHF formation in schizophrenia patients. The PON1 SNP variant was reported to provide a protective role; however, the genotypes, 192 QR and RR, are associated with higher levels of homocysteine [11], and significantly lower AREase activity was found in schizophrenic patients, along with significantly lower PONase activity in olanzapine-treated patients with the QQ genotype [12]. 

Though the analytical methodology reported 5-MTHF levels that were as low as 10 nM, according to a previous study, levels of 5-MTHF in healthy adults can be as low as 6 nM. It is necessary to develop a sensitive method that can be used to determine levels of 5-MTHF lower than 6 nM, since the levels can be even lower in schizophrenia patients. The R variant of PON1 [12] and the T variant of MTHFR C677T were associated with increased homocysteine levels [13], but whether the variants of PON1 and the variants of MTHFR were indirectly associated with reduced MTHF levels in schizophrenia patients is still unclear. Therefore, this study aimed to develop a quantitative system, and investigate the levels of 5-MTHF in schizophrenia patients and healthy controls carrying various PON1 Q192R and MTHFR C677T genotypes. Sex differences were also discussed.

## 2. Materials and Methods

### 2.1. LC-MS/MS

This study employed liquid chromatography–mass spectrometry (LC–MS/MS) coupled with high-performance liquid chromatography (HPLC; 1260 Infinity II Quaternary Pump LC system) and triple-quadrupole mass spectrometry (Agilent 6470 LC–MS/MS system, Agilent Technologies, Inc., Santa Clara, CA, USA), the results of which were analyzed (Agilent jet stream electrospray ionization, AJS ESI) [14], and multiple reaction monitoring (MRM) was used to repeatedly screen and detect fragments, and minimize the basal effect matrix effect. The negative voltages were set at 4000 and 3500, whereas the parameters of the nitrogen gas and sheath gas were set at 45.0 psi, running at 5.1 L/min or 11.0 L/min, and the temperature was set at 300 °C or 250 °C, respectively.

The extracted sample was separated via an analytical column (ACQUITY UPLC BEH Amide Column; L = 50 mm × 2.1 mm; 1.7 μm) after being run through a precolumn (ACQUITY UPLC BEH Amide VanGuard Precolumn; 5 mm × 2.1 mm, 1.7 μm while the temperature set at 30 °C). The mobile phase comprised solution A (10 mM ammonium acetate, pH 3.5) and solution B (acetonitrile, ACN) degassed with a sonicator. Initially, the run occurred using the following conditions: 15% solution A and 85% solution B at 0.5 mL/min for 10 min, which was equal to approximately 20× the column volume, to thoroughly ensure that the column was at equilibrium. The dried sample was reconstituted with solvent, and then each sample (5 μL) was eluted by gradient conditions for 7 min, which were set as follows: 0.0–0.5 min (15% A, 85% B), 0.5–1.5 min (15% A, 85% B–60% A, 40% B), 1.5–2.5 min (60% A, 40% B), 2.5–3.0 min (60% A, 40% B–90% A, 10% B), and 3.0–7.0 min (15% A, 85% B). The mass spectrum was set at positive ion mode and then analyzed by Agilent MassHunter Workstation^®^ Software to obtain the peak area of each analyte, and then to calculate the peak area ratio using the peak of the respective isotope molecule for further analysis.

A calibration curve was prepared after plasma from healthy donors was dialyzed in a 3.5 kD molecular weight cut off, MWCO Spectra/Por^®^6 Dialysis Membrane. After 26 h of dialysis to exclude the internal presence of 5-MTHF, the residual 5-MTHF in the dialyzed plasma was confirmed to have a lower response ratio than those of the lowest concentrations of STDs used for the standard curve. 5-MTHF stock solution was prepared with 1.0 mg in 9.93 mL 1% ascorbic acid/ddw, and then divided into 200 μL aliquots per tube and stored at −80 °C. Internal standard (IS) ^13^C5-5-MTHF (1.0 mg) was dissolved in 4.79 mL of 1% ascorbic acid/ddw, and ^13^C5-5-MTHF (60 μL) was diluted with 940 μL of 1% ascorbic acid/ddw. Then, ^13^C5-5-MTHF (150 μL) was stored at −80 °C. The working IS was then diluted again with 1% ascorbic acid to 2 μM, and 5 μL was added to each sample.

STD was prepared in ACN:MeOH (75:25) containing 1 g/L ascorbic acid to dilute the 5-MTHF stock into 2.5, 7.5, 15, 30, 60, and 80 nM. To generate the calibration curve, ACN:MeOH (75:25) containing 1 g/L ascorbic acid was used to prepare 5-MTHF at 40, 75, 200, 400, 600, 1000, and 1500 nM, and was then used for plasma extraction. For quality control tests, each 5-MTHF STD was further diluted into 2, 3.75, 10, 20, 30, 50, and 75 nM, and three different levels of 5-MTHF at 3.75 nM, 20 nM, and 50 nM were considered as the low-quality concentration (LQC), middle-quality concentration (MQC) and high-quality concentration (HQC), respectively.

### 2.2. Plasma Extraction

This study used the protein precipitation method to extract analytes. The detailed steps include the following: 200 μL plasma prepared in a microcentrifuge tube was added with 0.1 mg/L 2-ME and 0.1 mg/L ascorbic acid, and then, 5 μL of ^13^C5-5-MTHF (2 μM) was added, followed by 600 μL ACN:MeOH (1:2 containing 1 g/L ascorbic acid) as the extraction solvent. After vortexing rigorously, the precipitated proteins were separated by centrifugation at 10,500 rpm for 10 min at 25 °C. The supernatant was then evaporated with vacuum pressure under centrifugation (EYELA) and heated at 30 °C. Then, 100 μL ACN:MeOH (75:25, supplemented with 0.2% formic acid and 1 g/L ascorbic acid) was used as the reconstitution solvent and mixed thoroughly. Before loading them into the amber vial for LC–MS analysis, the samples were also passed through MS nylon syringe filters (0.22 μm, 4 mm).

Validation methods were adopted from the FDA Health and Services Guidelines (2018) [15] for the calibration curve, accuracy, precision, etc., when variations were within 15% of each concentration, except the one for the lower limit of quantification (LLOQ), which was within 20% of the theoretical values. Standard and calibration curves were determined using 6 different concentrations of standard solution either in the vehicle or spiked into the dialyzed plasma. The composition of the vehicle was ACN:MeOH (75:25) containing 1 g/L ascorbic acid, whereas ACN:MeOH (75:25) with 0.2% formic acid containing 1 g/L ascorbic acid was used as the reconstitution solvent. After analysis by LC–MS/MS, the peak areas of the standard or its IS were recorded, and each of the response ratios of various concentrations were then calculated to test the linear regression. The methodology was validated by a standard curve and STDs spiked into dialyzed plasma to test the accuracy, precision, recovery rate, and stability.

Accuracy and precision were tested at least 5 times for the low, middle, and high concentrations selected within the ranges from the standard curve. The mean variations were determined from the concentrations calculated from the calibration curve. Precision analysis used similar procedures, but was tested either within the same day as intraday precision or performed on five different days, which was considered interday precision. The determined concentrations calculated from the calibration curve were then expressed as their coefficient of variation (C.V.). The C.V. values for these accuracy and precision tests met the expected values (within ±15%), whereas the lower limit of quantification (LLOQ) (within ±20%) met the quality controls.

The recovery of 5-MTHF (3.75, 20, 50 nM) spiked in dialyzed plasma was calculated from five different batches of preparations according to the formula: (calculated peak area)/(peak area of respective concentration) × 100%. Stability was tested from a batch of tubes containing dialyzed plasma spiked with standards (STDs) at low, middle, and high concentrations within the standard curve ranges. After storage in −80 °C refrigerators for either 7, 28, 56, or 84 days, at least two tubes from the same batch were then analyzed for the analytes after extraction to compare the results with those obtained from the tubes that did not undergo refrigeration.

### 2.3. Clinical Assays

This study was approved by the IRB of Taipei Medical University (N201512021). Healthy controls were randomly selected from the Taiwan Biobank, and schizophrenia patients were recruited from Wan-Fang Hospital and Rehabilitation Institute. After receiving informed consent from patients or their families, patient medical records and history or physical examination records were reviewed; those who met the inclusion criteria were subjected to blood draws to separate plasma and the buffy coat for DNA extraction, and these samples were then stored at −80 °C for further analysis.

The criteria for schizophrenia patients, including their diagnosis, were based on the guidelines of the Diagnostic and Statistical Manual of Mental Disorders IV (DSM-IV-TR) [16]; the ages of patients ranged between 20–65 years old, and the exclusion criteria included those with psychiatric disorders, except schizophrenia; current substance abuse; medical conditions that may confound glucoregulatory assessment, including diabetes mellitus and other endocrine diseases; severe cardiovascular, hepatic, or renal disease; malignancy; epilepsy; pregnancy; or lactation.

DNA was extracted by a QIAamp DNA Blood Mini Kit from patient buffy coat samples or was obtained from biobank samples directly. The quality of DNA was determined with the UV spectrum at A260/280 (purity; 1.7~1.9) and at A260/230 (limited salt contamination; 2.0~2.4). The inlay nucleotide polymorphism genotyping (SNP genotyping) method adopted from Chen et al. [17], the TaqMan™ Genotyping Master Mix and TaqMan™ SNP Genotyping Assay using PCR instruments, and the reports were obtained by Applied Biosystems Sequence Detection Systems Version 2.4.1. or via TWB2.0 array to determine the SNP variants of MTHFR C677T (rs1801133) and PON1 (rs662).

### 2.4. Statistical Methods

Descriptive results, such as the mean ± standard deviation, percentage (%), and regression lines, were analyzed using Microsoft Excel 2016. A Hardy–Weinberg equilibrium (HWE) analysis of the SNP genotypic frequency was tested. A boxplot was applied for graphically depicting groups of numerical data through their quartiles to show the outliers. SPSS version 19 was used to determine data related to patients or healthy subjects, as well as statistical methods, such as the chi-squared test for measuring the association between two categorical variables, and the independent *t* test for testing differences between two different groups. Results with a *p* value of less than 0.05 were considered statistically significant.

## 3. Results

**Figure 1:**
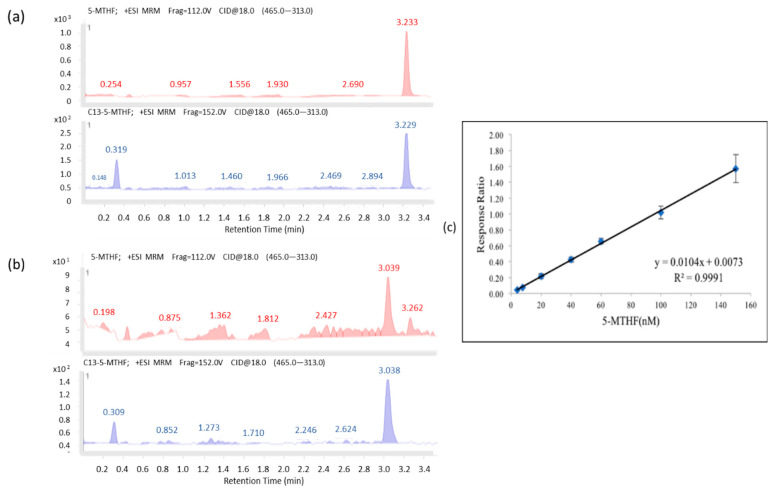
Chromatograms of 5-MTHF: (**a**) the standard (in pink; 40 nM 5-MTHF; retention time 3.233 min) and its spiked internal standard (in blue; ^13^C5-MTHF 96 nM; retention time 3.229 min) prepared in sample solvent: ACN: MeOH (75:25, containing 1 g/L ascorbic acid); (**b**) the extracted plasma sample spiked with spiked internal standard. LC programs were set at a flow rate of 0.5 mL/min with mobile phase A (10 mM ammonium acetate (pH 3.5)), mobile phase B (ACN 0.0–0.5 min (15% A, 85% B)) for 0.5–1.5 min (15% A, 85% B–60% A, 40% B), 1.5–2.5 min (60% A, 40% B), 2.5–3.0 min (60% A, 40% B–90% A, 10% B), and 3.0–7.0 min (15% A, 85% B); (**c**) Response ratio of the extraction curve for the 5-MTHF at concentration ranges of 4–150 nM.

As shown in Figure 1a,b, the chromatograms for 5-MTHF (*m/z* 460.0) and its internal standard, ^13^C5-5-MTHF (*m/z* 465.0), were fragmented into 5-MTHF (*m/z* 313.0) and ^13^C5-5-MTHF (*m/z* 313.0). The retention time for 5-MTHF was 3.277 min, whereas those for plasma 5-MTHF and its ^13^C5-5-MTHF spike-in were 3.233 and 3.229 min, respectively. The validation results are shown in Table 1 and Table 2. The concentration range was 4–150 nΜ, and the results of calibration regression were plotted by using the spiked 5-MTHF concentrations as the *x*-axis against the *y*-axis, which was calculated as the peak area vs. IS as the response ratio.

As shown in Figure 1c, the regression line of the calibration curve, y = 0.0104x + 0.0073 (R^2^ = 0.9991), with the corresponding relative standard deviation percentage (%RSD) values of 5.58–14.86% (the one for the lowest concentration (4 nM) was 15.78%) were all less than 15%, and no signal of >20% of the limit of detection (4 nM) at the respective retention time was found and showed adequate reproducibility, rendering the method acceptable. The recovery rate of 5-MTHF was 81.7–87.9%, and the stability of the STDs spiked in plasma stored at −80 °C was observed for up to 84 days (85.99~113.43%).

The validated analytical methodology was also successfully applied to 76 recruited subjects. As seen in Table 3, these pilot results show that schizophrenia patients had significantly lower (50%) levels of 5-MTHF (11.70 nM; n = 34) than those observed in healthy controls (22.67 nM; n = 42), and there was a significant difference in the frequency of MTHFR genotype variations, but not PON1 genotype variations, between the recruited schizophrenia patients and healthy controls.

**Figure 2:**
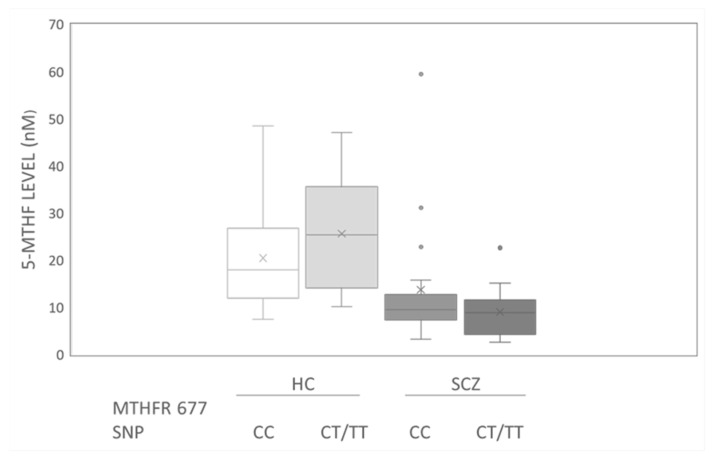
Median levels of 5-MTHF concentration in schizophrenia patients (SCZ; n = 34) vs. healthy controls (HC; n = 42), subgrouped by variants of MTHF677 polymorphisms. The outliers in schizophrenia are indicated as dots either in CC or CT/TT carriers. × markers indicate mean value of each group.

However, both within the schizophrenia patients and healthy controls, there were no differences in 5-MTHF concentration between those with CC and those with CT/TT, as shown in the box plots (Figure 2). As shown in Table 4 and Table 5, the mean plasma 5-MTHF levels in male and female schizophrenia patients were 10.21 and 13.38 nM, respectively, which were 40% lower than those in healthy subjects. After subgrouping by sex, the genotypes of MTHFR and PON1 were investigated. Male healthy controls with CT variants exhibited significantly lower levels of 5-MTHF than those observed in the female controls, mostly carrying Q alleles of PON1, as shown in Table 5. The mean levels of MTHF in the male schizophrenic group with CT variants were also determined to be 50% lower than those in female patients, but the difference was not statistically significant (Table 4). Moreover, in MTHFR TT subgroups, those exhibiting the presence of at least one R allele of PON1 variants had higher mean MTHF levels (34.31 or 28.65) than those without R alleles (12.08).

## 4. Discussion

The current study established an LC–MS/MS system coupled with an amide column, and first successfully applied it to determine the 5-MTHF levels in both schizophrenic patients and healthy controls in Taiwan. The analytical methodology can be used to quantify levels of 5-MTHF as low as 2 nM within 7 min because dialyzed plasma was employed to establish the calibration curve; this approach can be successfully applied to 200 μL of plasma to accurately quantify the lower 5-MTHF levels observed in schizophrenic patients by reconstitution with 100 μL of the vehicle before analysis. The results showed that patients exhibited significantly lower mean levels of 5-MTHF than healthy controls.

The levels of 5-MTHF were lower than 6 nM not only in six male patients (CC/CT = 2/4), but also in one healthy male control, which can be considered to represent nutritional insufficiency, based on the 5-MTHF levels in healthy adults (ranges: 6.6–39.9 nM) reported in a study conducted in Caucasians [18]. Such sex differences may also provide an explanation for the significant improvement in psychotic activity over 20 years that is observed in women, but not in men [19].

Several studies aimed to determine 5-methyltetrahydrofolic acid levels, but utilized different methods, as illustrated by the following examples. (1) A recent study [20] that did not exclude the effects of the endogenous levels reported that the LLOQ for 5-MTHF levels was 25 ng/mL (~55 nM), which was 20 times less sensitive than the LLOQ (2 nM) observed in this study, which also provided better sensitivity than the value observed in Zheng et al.’s [21] methodology (10.1 nM). (2) Some studies used reversed-phase analytical C18 columns, e.g., in the studies of Wang et al. [22] and Zheng et al. [21], whereas the other studies [23] used HILIC columns. Therefore, different columns were washed with different mobile phases and elution conditions; for example, 0.1% formic acid [23] was mixed with water and acetonitrile (ACN), whereas 1 mM ammonium acetate containing 0.6% formic acid was mixed with CAN as the mobile phase [21]. Unlike in previous studies, the HILIC column used by Garbis et al.’s [23] study used ACN:water (75:25) with 5 mM ammonium acetate, pH 6, in isocratic elution, and was reported to result in a better resolution of 5-MTHF peaks. (3) Regarding the stability of the methodology, the observed results showed that standard samples prepared in plasma were stably stored in a −80 °C freezer for at least 84 days. Such results support the observation of stability in Zheng et al.’s [21] study, in which they stored samples at a low temperature for 50 days. (4) Regarding the sample type, Hannisdal et al. [24] analyzed both plasma and serum samples, and the levels of 5-MTHF observed in the two were similar, at 16.8 and 16.5 nmol/L; however, 5-MTHF levels in serum were more stable than those in plasma stored at room temperature. Even though the 5-MTHF level was reduced faster in plasma [24], the sample evaporation or extraction process that occurred under 4 °C in this study minimized the extent of possible degradation.

Sex differences in 5-MTHF levels were observed in the current study. As seen in Figure 2, male healthy controls who carried MTHF CT/TT variants exhibited the highest median levels of 5-MTHF among these four groups, stratified by diagnosis with schizophrenia and T allele variants; conversely, the patient group exhibited the reverse trend. In fact, according to the results of Table 4, there was no patient identified as having the TT variant. This finding was considered together with the results of the analysis of male TT carriers in the healthy controls (Table 5), who exhibited 5-MTHF levels at 34.31 nM, which were 20 nM higher than the levels observed in those carrying CC/CT in the healthy male subjects (11.81~14.73 nM). It can be speculated that males carrying TT mutants may possess protective factors, resulting in beneficial levels of 5-MTHF or a lower incidence of developing schizophrenia.

In fact, the folate levels determined in Ni et al.’s [9] study also showed similar sex differences, since the levels in males (19.46 nM) were significantly lower than in female (22.99 nM) healthy volunteers. The factors contributing to the observed sex differences may result from the fact that dietary vitamin supplementation was more common in females [25], and may also be related to differences in smoking and drinking, which can result in lower intake or absorption of folic acid. Therefore, those who may have insufficient MTHF levels should not only be identified, but should also be considered for supplementation with folates. Willems et al. [26] recruited patients with cardiovascular disease with MTHFR C677T SNP variants, and followed the patients over a two-year period using a randomized crossover study to test the pharmacokinetics of oral 5 mg 5-MTHF or 5 mg folate. The results indicate that 5-MTHF had higher bioavailability, and the Cmax of 5-MTHF was seven-fold that of folate [26]. It was also suggested that 5-MTHF supplementation did not produce differences between patients with different SNP variants, and that the outcomes of this approach may be because it was used to improve homocysteine metabolism and the one-carbon metabolism cycles.

The benefit of supplementation with 15 mg 5-MTHF in patients with schizophrenia was also reported by Roffman et al. [27] to lead to lower total Positive and Negative Syndrome Scale (PANSS) scores and negative and general psychopathology scores after 12 weeks, compared to those observed in the placebo groups. Although the above studies did not monitor patients’ levels of folates, the current study data regarding 5-MTHF levels support the rationale that 5-MTHF can be used as a complementary supplement for patients with schizophrenia, even though there was no correlation of 5-MTHF to patients’ PANSS total scores, and no sex difference of total PANSS was observed. The differences in 5-MTHF levels between the schizophrenia group and healthy controls were approximately 10 nM, and whether 15 mg 5-MTHF is the optimum dose for the current study population warrants confirmation in future studies.

The lower mean levels of MTHF that were observed in male subjects with CT variants (Table 4 and Table 5), and the higher percentage of subjects who carried QQ of PON1 variants in the subgroups, who also had higher mean MTHF levels than those without R alleles, provide an explanation for the results of an Indian study demonstrating that genotypes of MTHFR C677T CT and PON1 Q192R QQ were significantly associated with the risk of coronary artery disease [28,29]. 5-MTHF is produced by MTHFR, and 5-MTHF assists in remethylation of the homocysteine produced by the PON1 enzyme from its precursor (homocysteine-thiolactone). In addition to the levels of 5-MTHF, the levels of the other metabolites involved in one carbon cycle require further investigation in a larger population in Taiwan to develop guidelines to improve health in the general population and schizophrenia patients. Therefore, the potential uses of current analytical methods to measure the other carbon metabolites simultaneously were also assessed during their development. In particular, to avoid the analyte oxidation observed under room temperature [22] or in a warm bath [19] during dryness, the current study employed not only simple vacuum centrifugation in a cold environment at 4 °C to dryness after extraction, but also tried to use different ratios of ACN:MeOH with various percentages of ascorbic acid to extract analytes and different formulas of the reconstitution solvent [30,31] in healthy subjects. The value of the current extraction and reconstitution processes is that they have also been applied in quantifying one carbon metabolites successfully in some schizophrenic patients [32], but not all patients, due to the large differences between betaine and 5-MTHF. The concentration ranges of the calibration curve may need to be adjusted [32] to include all tested samples within the concentration ranges of various metabolites.

A limitation of the current study is that this is the first pilot study in Taiwan to apply validated analytical methods for investigating the levels of MTHF in small samples of subjects together with SNP identification for PON1 (rs622) and MTHFR (677). The current study only recruited two females, but no male schizophrenic T carriers of MTHFR C677T, which may account for no differences of MTHF levels in comparison to CC carriers. The individual therapy prescriptions and dietary differences may also influence the measured results. In the future, the prevalence of insufficient MTHF levels or the frequency of genotype variations in different SNP sites of MTHFR [33] or PON1 [34] in larger populations, including in healthy persons, are needed to clarify the roles of the SNPs of enzymes involved in patients’ symptom scores and one-carbon metabolism cycles, other than PON1 and MTHFR, in regulating 5-MTHF concentrations and the other one-carbon metabolites, such as betaine and choline.

## 5. Conclusions

This study developed an analytical methodology with a fast, stable, and simple process with better sensitivity, and minimized the residues of internal 5-MTHF in dialyzed plasma to produce a reliable calibration curve. Schizophrenia patients exhibited mean levels of 5-MTHF that were 50% less than those in the healthy controls, especially in male patients with CT variants (38%). Among healthy subjects, significantly lower levels of 5-MTHF were also seen in male subjects. Carrying MTHFR CT/PON1 Q alleles may be a negative factor on plasma 5-MTHF levels. Investigations in a larger population are warranted to confirm the observed sex or gene polymorphism effects.

## Figures and Tables

**Table 1:** Precision of the 5-methyltetrahydrofolate (5-MTHF) standard and extraction calibration curve.

5-MTHF (n = 5)	Ranges (nM)	Concentration	Precision RSV (%)
Standard curve	2.5–80	2.5	4.32
		7.5	4.44
		15	9.19
		30	10.22
		60	9.27
		80	7.81
Calibration curve	4–150	4	15.78
		7.5	6.67
		20	14.86
		40	7.48
		60	5.58
		100	7.93
		150	11.25

**Table 2:** Validation of interday and intraday analysis of the 5-MTHF spike in dialyzed plasma.

Spike Conc.	Calculated Conc.	Accuracy (%)	Precision RSV (%)
	Intraday Analysis (n = 5)		
7.5	7.38 ± 0.62	98.39	7.63
40	45.38 ± 3.66	113.45	7.94
100	97.99 ± 6.56	97.99	6.64
	Interday analysis (n = 5)		
7.5	7.11 ± 0.67	94.80	8.55
40	40.65 ± 4.50	101.63	10.89
100	100.44 ± 4.74	100.44	4.68

**Table 3:** Demographics of schizophrenia patients vs. healthy controls.

	Healthy Controls (n = 42)	Schizophrenia Patients (n = 34)	*p* Value
Age, mean ± SD	44.67 ± 10.54	45.91 ± 10.63	0.61
Sex M/F	14/28	18/16	
5-MTHF (nM)	22.67 ± 11.12	11.70 ± 10.37	<0.01
PON1 Q192R (rs662)	HWE *p* = 0.47	HWE *p* = 0.56	0.56
QQ, n (%)	15 (35.7)	12 (35)	
QR, n (%)	22 (52.4)	15 (44)	
RR, n (%)	5 (11.9)	7 (21)	
MTHFR C677T	HWE *p* = 0.05	HWE *p* = 0.90	<0.001
CC, n (%)	24 (57.1)	19 (55.9)	
CT, n (%)	12 (28.6)	13 (38.2)	
TT, n (%)	6 (14.0)	2 (5.9)	

**Table 4:** Sex differences in 5-MTHF levels in schizophrenic patients with different patterns of MTHFR C677T and PON1 variants.

Schizophrenia	M (n = 18)		F (n = 16)	
Age	44.89 ± 12.35		47.06 ± 8.55	
5-MTHF (nM)	10.21 ± 12.66		13.38 ± 7.00	
MTHFR C677T	MTHFR levels (N)	No. of PON1 variants (QQ/QR/RR)	MTHFR levels (N)	No. of PON1 variants (QQ/QR/RR)
CC	13.70 ± 16.28 (n = 10)	5/2/3	13.95 ± 8.36 (n = 9)	1/7/1
CT	5.84 ± 3.08 (n = 8)	4/3/1	12.86 ± 6.12 (n = 5)	0/3/2
TT	no patient		12.08 ± 4.43 (n = 2)	1/0/0

**Table 5:** Sex differences in 5-MTHF levels in healthy controls with different patterns of MTHFR C677T and PON1 variants.

Healthy Controls	M (n = 14)		F (n = 28)	
Age	43.28 ± 10.96		45.36 ±10.46	
5-MTHF (nM)	18.30 ± 10.37 *		24.83 ± 11.01	
MTHFR C677T	MTHFR levels (N)	No. of PON1 variants (QQ/QR/RR)	MTHFR levels (N)	No. of PON1 variants (QQ/QR/RR)
CC	14.73 ± 10.13 (n = 8)	4/2/2	23.30 ± 10.14 (n = 16)	5/9/2
CT	11.81 ± 1.43 * (n = 3)	0/3/0	26.69 ± 13.37 (n = 9)	5/4/4
TT	34.31 ± 5.36 (n = 3)	1/2/0	28.65 ± 10.02 (n = 3)	1/2/0

* *p* < 0.05 in comparison to female subjects with the same genotype by *t*-tests.
